# Previous trauma exposure and self-mastery as moderators of psychiatric effects of home isolation during the Covid-19 pandemic: a field study

**DOI:** 10.1186/s12888-022-04087-8

**Published:** 2022-07-05

**Authors:** Michelle Slone, Ayelet Pe’er, Flora Mor

**Affiliations:** 1 School of Psychological Sciences, Tel Aviv University, 69978 Tel Aviv, Israel; 2 JDC Ashalim-Israel, Eliezer Kaplan St 9, P.O.B.3489, 9103401 Jerusalem, Israel

**Keywords:** Covid-19, Home isolation, Mastery, Psychiatric symptoms, Resilience, Trauma

## Abstract

**Background:**

Limiting contagion during the Covid-19 pandemic has necessitated employment of drastic measures ranging from complete lockdown to home isolation and quarantines. This study examined the psychiatric effects of home isolation, the effects of interacting previous traumatic events and the moderating effect of self-mastery as a resilience factor that could mitigate negative effects.

**Methods:**

Six hundred forty-five adults aged 18–67 completed an online survey during the first wave lockdown during the Covid-19 outbreak in Israel. Participants completed a demographic questionnaire including measures of strictness of adherence to home isolation, a traumatic life events measure, the Mastery Scale, and the Brief Symptom Inventory. Data was analyzed using Structural Equation Model.

**Results:**

Findings showed positive relations between strictness of home isolation adherence and psychiatric symptoms, and between previous trauma exposure and psychiatric symptoms. A negative relation between self-mastery and psychiatric symptoms emerged. During home isolation, effects of previous trauma exposure on psychiatric symptoms was moderated by self-mastery. Individuals with high self-mastery showed less psychiatric symptoms than those with low self-mastery, at both high and low levels of previous trauma exposure.

**Conclusions:**

Home isolation adherence is associated with significant psychological distress and symptomatology and, thus, should be of great concern for public mental health service providers. The present study offers a new slant on appropriate clinical interventions during this period with a focus on strengthening resilience factors that can moderate mental health decline. Therapy and interventions based on promoting self-mastery could exert a significant effect on lowering psychiatric symptoms during stressful periods of home isolation.

**Trial registration:**

Not relevant.

The Covid-19 pandemic has struck the world with enormous impact in almost every sphere of life from the environmental, community, societal, family to the personal levels. The powerful infectious nature of the virus and high morbidity and mortality rates worldwide [1] has necessitated the employment of drastic measures to limit close contact. The forefront of disease containment measures has ranged from complete national or regional lockdown to home isolation and quarantines across most countries in the world [2]. Although lockdown conditions have varied across countries, most have typically involved home confinement and restrictions on non-essential public activities [3]. These socially and personally restrictive conditions have been correlated with increased psychological distress [2, 4] and negative emotions such as fear, uncertainty, and confusion [5, 6].

The multifarious psychological implications of the new life circumstances involved in the pandemic are now beginning to be understood, particularly the psychological ramifications of lockdown and home isolation. Comprehension of psychological effects is essential to inform and address public mental health related decisions and policies. In light of this significance, the present research examined the mental health effects of home isolation during the month-long lockdown in Israel from April 8^th^ to May 4^th^ 2020. Specifically, the study examined the effects of home isolation on psychological and psychiatric symptoms, the effects of the accumulation of previous traumatic events exposure together with home isolation on symptoms, and the possible interaction of a resilience factor, namely self-mastery, in mitigating negative effects.

## Effects of home isolation

Quarantine and home isolation mandates have generated serious concerns about the negative mental health effects of social isolation and loneliness [7]. Prolonged social isolation, defined as the lack of interactions with others [8], may range from complete social isolation with no or very little social contact to home isolation with restricted physical social contact and with or without interpersonal connections through virtual means. Social isolation is known to have alarming short-term and long-term psychological and psychiatric effects [2]. Enforced isolation and quarantines during previous epidemics have been linked to loneliness and mental health problems [9]. Indeed, mandates to self-isolate during the pandemic in the US led to a significant increase in self-reported loneliness, depression and suicidal ideation [10].

Emerging literature during the epidemic shows the development of a variety of psychological disorders such as anxiety, panic, obsessive–compulsive symptoms, insomnia, digestive problems, depression and post-traumatic symptoms [11]. In a cross-nation study of the association between social isolation and mental health among 13,660 older adults using a global online survey, findings confirmed that social isolation is positively associated with psychological distress, although this association varied across countries [12]. An interesting longitudinal study of the effects of confinement in Spain sampled participants at the beginning of the lockdown, one month later and at the lifting of the lockdown [13]. Results revealed sharp increases in depressive symptoms, anxiety and post-traumatic stress as the confinement progressed, with improvement as restrictions eased, but no return to pre-crisis levels. The accumulation of all these findings point to the mental health toll suffered in national attempts to contain contagion, emphasizing the traumatic nature of the pandemic and the necessity for clinical intervention strategies to mitigate psychological distress.

## Resilience

While the negative effects of the pandemic as well as other traumatic experiences are widely documented, studies have shown that even after severely traumatic events, individuals may show surprising resilience [14–16]. This evidence supports the conceptualization of the presence of resilience factors as a protective process that alleviates the consequences of risk exposure [17]. Accordingly, wide individual variations between previous trauma experiences and psychological response have been demonstrated across many types of trauma [14, 18].

Much research has contended that individual characteristics can represent risk and protective factors that are integral to the daily stress-and-coping process [19, 20]. Risk factors are associated with vulnerability and increased likelihood of negative outcomes [21], whereas protective or resilience factors are variables or processes that lead to decreased probability of negative outcomes or increased probability of better-than-expected outcomes [22].

During the Covid-19 epidemic, some fledgling research has begun to emerge regarding resilience factors. A study conducted in Japan showed a significant effect for ego-resiliency on reduction of depression and stress levels during the epidemic [23]. Additionally, in a study conducted in Spain during the mandatory home isolation, results showed that vigorous physical activity was associated with higher resilience in terms of greater locus of control, self-efficacy, and optimism [24]. Undoubtedly, further research is crucial to disentangle resilience factors that can mitigate the psychological distress associated with social isolation and offer an avenue to the development of appropriate interventions.

Resilience factors can be conceptualized as variables that moderate between trauma exposure and psychological outcomes [18]. According to this model, given exposure to trauma, a resilience factor would be any variable whose presence at high levels mitigates the expected negative effects of trauma exposure, whereas its non-existence or presence at low levels is associated with negative outcomes. This model has been translated into a useful research paradigm in several studies investigating the risk or protective function of a variety of personal, familial, community and environmental factors in dealing with traumas such as war, armed conflict, terrorism, and indirect media exposure to traumatic events [14, 25, 26].

## Self-mastery

In line with the above model of resilience, the present study examined the role of self-mastery as a resilience factor that could moderate between the experience of home isolation and quarantines during the Covid-19 pandemic and mental health outcomes. Self-mastery seemed a likely candidate to function as a resilience factor in view of previous research demonstrating the protective effects of high self-mastery in a variety of traumatic circumstances [19, 27].

Self-Mastery refers to a sense of having control over life events [28] and is reflected in a self-perception of strength and the capacity to cope with and overcome obstacles by relying on personal efforts [29]. Strong self-mastery is considered a central management resource in many models of stress [30] that appears to operate by maximizing the use of other resources and actions that promote the achievement of personal goals [31]. Studies have shown an association between strong self-mastery and lower levels of anger and depressive moods [32] and less negative affect [33] and higher levels of positive affect [34]. High self-mastery, together with other positive qualities such as optimism, vitality and positive affect, have been correlated with increased coping with disability among the elderly and lower rates of mortality among the elderly [35].

In its original conceptualization, self-mastery is described as a modifiable trait that can open avenues for therapies [36], thus offering potential implications for clinical intervention. In clinical populations, self-mastery have been found to predict higher self-ratings of health, greater sense of capability and competence, and greater improvements in depression [37, 38].

## The present study

In order to disentangle some of the aspects of the Covid-19 pandemic as a form of mass trauma associated with major psychological implications on the population, the present study investigated the effects of home isolation on psychiatric symptoms and the role of the accumulation of home isolation together with previous traumatic events on psychiatric symptoms. In addition, the study examined self-mastery as a candidate resilience factor that could mitigate negative psychological effects of home isolation and previous trauma exposure. In view of the above, four hypotheses were proposed.

## Hypotheses

The first hypothesis predicted a direct positive relation between the strictness of adherence to home isolation and psychiatric symptoms.

The second hypothesis predicted a positive relation between previous traumatic life events and psychiatric symptoms.

The third hypothesis predicted a negative relation between self-mastery and psychiatric symptoms.

The fourth hypothesis predicted that under conditions of isolation, effects of exposure to previous life events on psychiatric symptoms will be moderated by high self-mastery.

## Method

The aim of this study was to examine the psychiatric effects of home isolation during the Covid-19 pandemic. Potential moderating factors of self-mastery and previous traumatic events were examined as well. Due to the home isolation regulations, participants completed the study questionnaires online from their homes.

### Participants

Participants in the study were 645 Israeli adults, 57% women and 43% men, aged 18–67 (mean age 38.88), surveyed during the lockdown at the peak of the first wave of the Covid-19 outbreak in Israel in April 2020. The Israeli government declared a nationwide rigid lockdown during which people were required to stay secluded at their homes for over a month. Participants were recruited by online invitation via a Research Online Survey Service. All participants completed the online survey on the Qualtrics platform from their homes while in home isolation. Participant demographics included marital status (24% single, 67.8% married, 8.2% divorced), socio-economic status (6.8% low, 78.9% average, 14.3% high), and educational level (40.2% completed high-school education, 33.5% Undergraduates, 16.7% Postgraduates, 9.6% other). Four participants did not complete all the questionnaires and, therefore, these respondents were excluded from the study. A demographic questionnaire was compiled in line with the unique circumstances and included questions regarding the specific conditions of home isolation.

### Instruments

#### Mastery scale

Self-mastery was assessed with the Mastery Scale [28] which measures respondents’ general perceived control over life circumstances. Participants are requested to rate seven items (e.g., “I have little control over the things that happen to me”) on a Likert scale, from 1 (not at all) to 7 (very much) such that high scores indicate high levels of self-mastery. High test–retest reliability of at least 0.85 and satisfactory internal reliability levels (a = 0.75) have been reported for the scale [39]. In the present study, internal reliability was α = 0.80.

#### Traumatic life events measure

In the current study, the traumatic life events scale was comprised of the severe trauma events appearing in the Major Life Events Questionnaire [40], such as death of a close friend or relative, divorce, and having an illness or accident requiring hospitalization, with the addition of relevant items from the Political Life Events (PLE) Scale [41] regarding exposure to armed conflict events. Since the study was conducted in Israel, where citizens have been exposed to armed conflict events for decades, seven items of the PLE were included, such as being a victim of a terror attack or witnessing gunshots or the use of other weapons or explosives. Participants rated the impact of all experienced events on a Likert scale of 1 (very little impact) to 5 (very high impact) to produce a summed score. There is no theoretical rationale for calculating an internal consistency score for life events questionnaires, since there is no reason to expect consistency in experiencing discrete events [42]. However, high test–retest reliability has been reported for both the Major Life Events scale (*r* = 0.72), [40]and the PLE (*r* = 0.86 to *r* = 0.94), [42]. In addition, for the Major Life Events scale, self-reports have been found to be highly consistent with interview-based ratings, *r* = 0.89 [40].

#### Home isolation

Although national regulations restricted the population to home isolation, variations existed in permitted strictness of adherence. Participants were asked to report their strictness of adherence to home isolation on a scale of 1 (essential services—individuals who left home to work in essential services), 2 (partial isolation—individuals who left home to conduct essential tasks such as shopping for food or medication), or 3 (complete home isolation).

#### Brief Symptom Inventory (BSI)

Psychiatric symptoms were assessed using the Brief Symptom Inventory (BSI; 41) which comprises 53 self-report items rated on a Likert scale ranging from 0 (not at all) to 4 (very much). The measure yields a global severity index (GSI) indicating general psychological distress, calculated as the average of all symptoms. In addition, the scale yields 10 symptom subscales – anxiety, depression, somatization, obsessive–compulsive, interpersonal sensitivity, hostility, phobic anxiety, paranoid ideation, psychotic ideation, and one miscellaneous subscale. Items include “feelings of worthlessness”, “feeling fearful”, “nervousness or shakiness inside”, “temper outbursts that you cannot control”. In the present study, 9 of the 10 subscales were calculated, excluding the short miscellaneous subscale which includes discrete items. Good reliability and validity measures have been reported for the inventory (α = 0.71 to 0.81) as well as high test–retest reliability (correlations between 0.78 to 0.90) and high concurrent validity with the Minnesota Multiphasic Personality Inventory (MMPI; 42). In the current study, the BSI yielded Cronbach’s α coefficients of 0.81 for anxiety, 0.86 for depression, 0.86 for somatization, 0.81 for obsessive–compulsive symptoms, 0.83 for interpersonal sensitivity, 0.72 for hostility, 0.75 for phobic anxiety, 0.73 for paranoid ideation, and 0.74 for psychotic ideation.

### Procedure

After receiving authorization from the Tel-Aviv University Ethics committee, respondents received an online invitation to participate in the study from a Research Online Survey Service and, after providing written consent, they were provided with a link to the survey. Exclusion criteria were age below 18 years and incomplete responses to all questionnaires. All participants included in the study completed the full battery of questionnaires that included a demographic questionnaire, the Mastery Scale, Traumatic Life Events Measure, and the BSI. All measures have been translated into Hebrew and are widely used in Israel.

### Statistical analyses

Analyses were conducted by transferring the data to SPSS26 and AMOS software. The statistical analysis used in the present study, was Structural Equation Modeling. Studies have shown that the subscales of the BSI are differentiated and informative as separate measures of different pathologies [43, 44] and that together they reflect a general factor of psychological distress [45]. The use of SEM allows the formation of a latent variable comprised of the nine subscales which reflects general distress. Descriptive statistics are presented as Means and Standard Deviations (SD) or rate (%). Structural Equation Modeling (SEM) was conducted using AMOS software. Goodness of fit and the significance of the effects were examined. Accordingly, indices such as Chi-square Mean/Degree of Freedom (CMIN/DF), Root Mean Square Error of Approximation (RMSEA), Comparative Fit Index (CFI), Goodness of Fit Index (GFI) and Adjusted Goodness of Fit Index (AGFI) were used. The dependent variable was general distress and the independent variables were home isolation, previous traumatic life events exposure and self-mastery. To examine the moderating effect of previous traumatic events exposure and self-mastery, the interaction of these two variables was included in the model as well.

## Results

### Descriptive statistics

The following table presents average ratings and standard deviations for each subscale of the Brief Symptom Inventory which is valuable as a descriptive indicator of severity of pathology and psychological distress during Covid-19 home isolation regulations.

### Pearson correlations of study variables

Table 1 presents Pearson correlations between the study variables. Results show that while partial isolation was positively associated only with depression, complete isolation was positively associated with all nine pathology sub-scales. Additionally, as expected, self-mastery was negatively associated with all nine pathology sub-scales, and previous traumatic events score was positively associated with all sub-scales of psychiatric symptoms. Finally, the nine pathology sub-scales were highly associated with each other.

**Table 1:** Pearson correlations between study variables (*N* = 645)

	1	2	3	4	5	6	7	8	9	10	11	12	13	14
1. Partial Isolation	-													
2. Complete Isolation	-.48^**^	-												
3. Self-Mastery	-.01	-.01	-											
4. Previous Trauma Events	.04	.01	-.12^**^	-										
5. Anxiety	.04	.07^*^	-.40^**^	.18^**^	-									
6. Depression	.07^*^	.07^*^	-.45^**^	.17^**^	.76^**^	-								
7. Paranoid Ideation	-.01	.11^**^	-.42^**^	.18^**^	.68^**^	.63^**^	-							
8. Somatization	.00	.12^**^	-.34^**^	.26^**^	.71^**^	.65^**^	.56^**^	-						
9. Phobic Anxiety	.01	.13^**^	-.32^**^	.08^*^	.73^**^	.58^**^	.54^**^	.59^**^	-					
10. Hostility	.03	.09^*^	-.39^**^	.13^**^	.68^**^	.63^**^	.60^**^	.55^**^	.46^**^	-				
11. Psychotic Ideation	.03	.08^*^	-.44^**^	.18^**^	.76^**^	.82^**^	.70^**^	.69^**^	.62^**^	.62^**^	-			
12. Obsessive Compulsive	.02	.11^**^	-.39^**^	.22^**^	.73^**^	.73^**^	.68^**^	.67^**^	.60^**^	.61^**^	.75^**^	-		
13. Interpersonal Sensitivity	.00	.10^**^	-.37^**^	.19^**^	.70^**^	.73^**^	.73^**^	.59^**^	.53^**^	.63^**^	.76^**^	.69^**^	-	
14. General Distress	.02	.12^**^	-.47^**^	.21^**^	.90^**^	.87^**^	.81^**^	.80^**^	.77^**^	.75^**^	.90^**^	.86^**^	.85^**^	-

^*^ *p* < .05, ** *p* < .01

Note. Partial isolation and complete isolation are dummy variables (0 = No, 1 = Yes)

### Structural Equation Modeling (SEM)

**Fig. 1:**
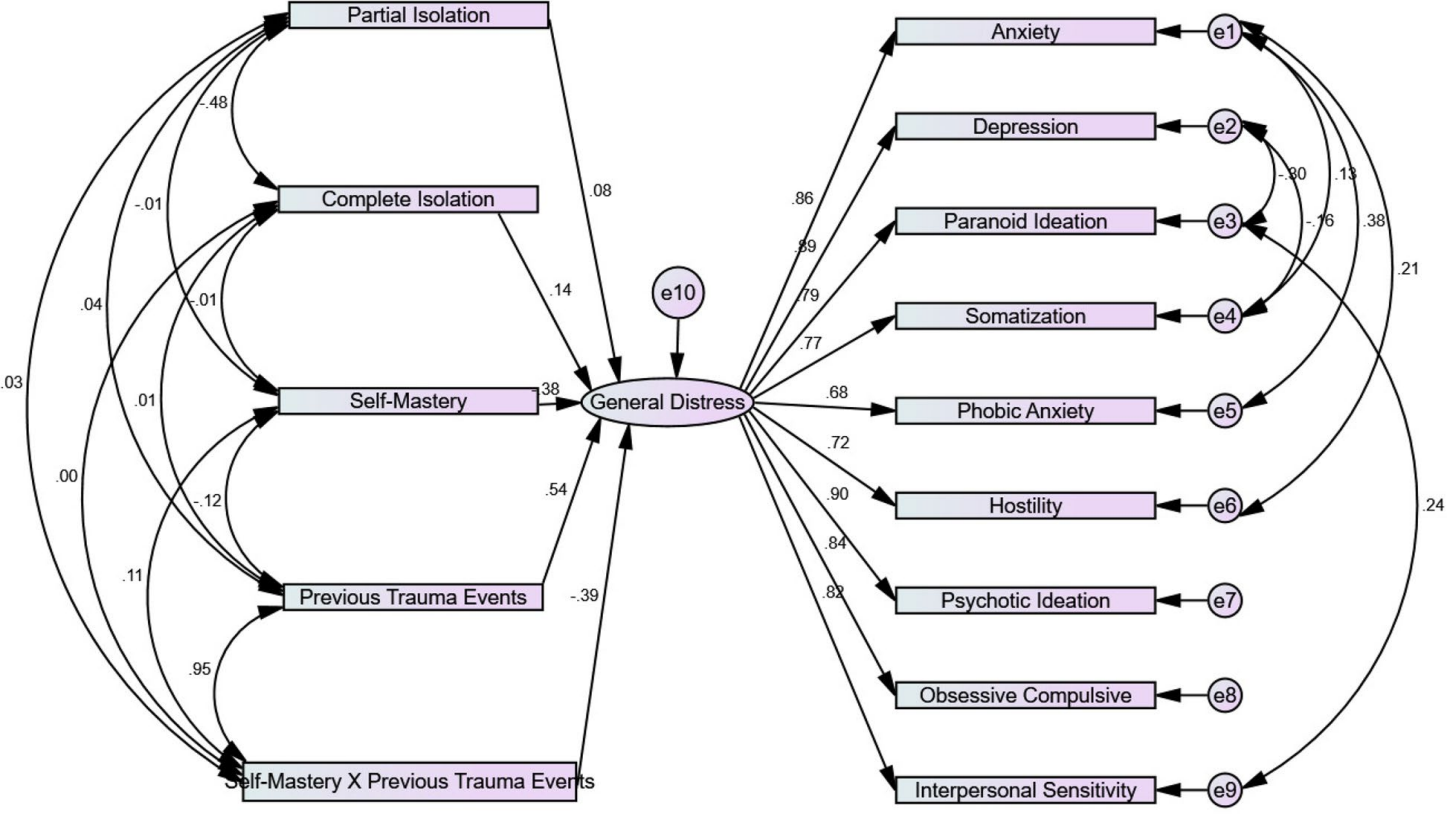
Structural equation model of home isolation, previous trauma events, self-mastery and the interaction between self-mastery and previous trauma a predictors of general distress. Note. The coefficients represent on the figure are standardized. Goodness of fit indices: χ 2 (61, *N* = 645) = 2.42, *p* < .001, GFI = .97 (AGFI = .95), CFI = .988, RMSEA = .047

The study hypotheses were tested using a Structural Equation Model (See Fig. 1). The model yielded acceptable goodness of fit indices: ${\chi }^{2}$ (61, *N* = 645) = 2.42, *p* < 0.001, *GFI* = 0.97 (*AGFI* = 0.95), *CFI* = 0.988, *RMSEA* = 0.047. Results showed that all nine sub-scales of psychiatric symptoms loaded highly on the latent variable of general distress ($\beta$ *s* ≥ 0.68). The first hypothesis, predicting a direct relation between home isolation and general distress, was confirmed as partial isolation ($\beta$ = 0.08) and complete isolation ($\beta$ = 0.14) both positively predicted general distress. The second hypothesis, predicting a direct relation between previous trauma exposure and general distress, was confirmed as well. Greater traumatic exposure positively predicted general distress ($\beta$ = 0.54). The third hypothesis, which predicted a direct negative relation between self-mastery and general distress, was also confirmed, ($\beta$ = -0.38).

The fourth hypothesis predicted that under conditions of home-isolation, self-mastery will moderate the effects of exposure to previous life events on general distress. This hypothesis was confirmed as well, as a significant moderating effect of self-mastery on the relation between previous trauma exposure and general distress emerged ($\beta$ = -0.39). The negative relation between the interaction variable and general distress reflects that the higher the self-mastery score, the more negative (i.e., less positive) is the relation between previous trauma events and general distress.

Examination of the interaction pattern revealed that for those with low self-mastery, the relation between previous trauma events and general distress was positively significant ($\beta$ = 0.21, *p* < 0.001), as expected. However, for those with high self-mastery, the relation between previous trauma exposure and general distress was non-significant ($\beta$ = 0.06, *p* > 0.05). This means that self-mastery acts as a moderating factor, as the higher the level of self-mastery, the weaker the relation between previous trauma exposure and general distress.

## Discussion

The first hypothesis predicted a direct positive relation between strictness of adherence to home isolation and psychiatric symptoms. This hypothesis was confirmed. The highest level of strict adherence to home isolation was defined as complete seclusion in the home and this category ranged from lone residence in the home or room isolation in institutionalized residential settings to home isolation with the nuclear family. This finding concurs with research evidence showing that social isolation during mandates for home isolation and quarantines can be emotionally debilitating, associated as it is with loneliness and mental health difficulties such as depression, anxiety and post-traumatic stress [2, 7].

The unexpected pandemic has spread fear, anxiety, and a sense of insecurity and danger among populations worldwide. Results of this study show that levels of psychiatric symptoms were high across all three levels of home isolation. As can been seen in Table 2, the average ratings for the BSI subscales in the present study range from 1.045 to 1.834 which reflect high levels of psychological distress and symptom profiles. These ratings far exceed the Israeli norms for the BSI which range from 0.46 to 0.94 and are, in general, higher than the US and British norms for the subscales [46]. Descriptive statistics suggest that partial isolation was directly associated only with depression, whereas complete isolation was directly associated with all nine sub-scales of psychiatric symptoms and general distress. Notwithstanding these significant associations, it should be noted that individuals in the complete home isolation group may or may not have engaged in social interactions by digital means, which became very popular during lockdown.

**Table 2:** Levels of Psychiatric Symptoms

	**Anxiety**	**Depression**	**Paranoid ideation**	**Somatization**	**Phobic anxiety**	**Hostility**	**Psychotic ideation**	**Obsessive–compulsive**	**Interpersonal difficulties**
Mean	1.819	1.754	1.545	1.387	1.834	1.045	1.532	1.650	1.469
SD	.691	.736	.609	.578	.802	.425	.612	.683	.702

In Israel, psychological distress may be generally elevated due to the prolonged armed conflict circumstances, however, the present ratings indicate an even higher increase in distress although virtually no hostilities occurred during the initial period of the outbreak of the epidemic. This highlights the enormous impact on public health of the Covid-19 pandemic on the Israeli population and its accompanying forced life changes. Similarity in strategies of home isolation employed globally suggest that these elevated levels of a wide variety of distress symptoms may apply to millions of people worldwide.

The second hypothesis predicting a positive relation between pre-isolation trauma exposure and psychiatric symptoms, was confirmed. This aligns with previous research on the negative impact of cumulative trauma exposure on emotional status [18, 19]. This finding is disturbing since it possibly reflects the deleterious effects of the cumulative trauma of previous traumatic exposure in addition to dealing with the pandemic outbreak crisis. Descriptive statistics suggest that higher levels of pre-isolation trauma exposure were related to all sub-scales of psychiatric symptoms and to general distress. The persistence of a full range of emotional difficulties as the pandemic progresses warrants further investigation and emphasizes the importance of identifying possible resilience factors that could mitigate these effects.

The third hypothesis predicting a negative relation between self-mastery and psychiatric symptoms was confirmed and this result aligns with previous studies indicating that strong self-mastery is associated with lower levels of depression [29] and negative affect [33]. Self-mastery has been found to be negatively associated with post-traumatic symptoms in studies of community disasters and armed conflict [19, 30]. Self-mastery and belief in personal capability to solve problems and execute actions required to manage life situations [47] would be a vital factor in coping with a the Covid-19 pandemic. Coping with fundamental life-style changes and habits, restrictions in freedom of movement, and altered working conditions necessitates taking some control over the situation by problem-solving, creativity, innovation and flexibility. These capabilities are embodied in strong self-mastery. Self-mastery could promote perceptions of traumatic experiences as an opportunity to develop strengths and undertake new modes of action [31]. Thus, proactive self-mastery over the new and challenging situation could go a far way in allaying depression, hopelessness, anxiety, and other post-traumatic symptoms.

The fourth hypothesis predicted that under conditions of home isolation, effects of previous trauma exposure on psychiatric symptoms will be moderated by high self-mastery. This hypothesis was also confirmed. Individuals with high levels of self-mastery showed lower levels of psychiatric symptoms than those with low levels of self-mastery, at both high and low levels of previous trauma exposure. For individuals with low levels of self-mastery, those with high levels of previous trauma exposure showed higher levels of psychiatric symptoms than those with low levels of previous trauma exposure. This interaction indicates that, under conditions of isolation, self-mastery alleviates the psychiatric effects of previous trauma exposure.

The moderating effect of self-mastery could be explained by the experience of having more control over life events [28] and greater ability to rely on personal efforts and to cope with challenges and adversities [29]. These abilities could explain the important function of self-mastery as a resilience factor specifically during the pandemic which has spread much uncertainty and fear. The pandemic has necessitated adaptation to different life-styles, changes in daily routine, re-organization of work places and schedules, the development of complex family dynamics, and different modes of social interactions. High self-mastery enables the search for new and innovative problem-solving strategies together with maintenance of feelings of autonomy and self-efficacy. The finding that high self-mastery moderates these symptoms has important clinical implications. Therapy and interventions based on promoting self-mastery could exert a significant effect on lowering these distressing symptoms.

## Limitations and conclusions

This study was conducted against a scarce background of research literature on the role of resilience factors in coping with Covid-19 induced stress, necessitating new comprehensions and conceptualizations of the subject at hand. Multiple challenges were involved in conducting the study including recruitment of participants during the lockdown, conceptualizing the pandemic as a new form of trauma, and selection and construction of appropriate measures. There are limitations of bias involved with non-random referral sampling and online sampling methods. Although a generous number of participants were successfully recruited, further research is needed to extend generalization to different populations sectors in Israel for whom isolation may be perceived differently, such as the elderly, individuals without access to the internet or digital technologies, and to other cultures. Additionally, cross-sectional research cannot determine causal relations between variables and studies of the moderating role of resilience factors should be augmented with longitudinal designs. Further, the study was based on single agent self-report and, therefore, should be extended to include multiple agent reports. The home isolation variable was based on a single direct question regarding the level of adherence to home isolation. It would be valuable to expand this measure and to assess levels and types of social contact maintained during lockdown, as well as violations of restrictions and methods of violation.

In addition, this study is limited by its narrow focus on a single potential resilience factor, namely, self-mastery. However, the present study is important in highlighting the benefits of promoting a general resilience factor, in this study self-mastery, rather than focusing on the pathology itself. Results suggest that enhancing self-mastery could prove to be a central therapeutic avenue for dealing with emotional distress raised by the pandemic.

A wide range of other potential resilience factors could be valuable moderators of the psychological distress and psychiatric symptoms associated with the Covid-19 pandemic. Further research should examine a long list of personal, family, and social factors that can mitigate psychological and psychiatric symptoms associated with the pandemic and the stressful circumstances that have arisen in its wake.

The present study could have far-reaching implications. The weakness imbued in examining a sudden, unexpected and worldwide phenomenon could also be construed as a strength in opening understanding of a new form of trauma about which little was heretofore known. The uniqueness and severity of the situations that have emerged with the Covid-19 pandemic necessitate rethinking therapeutic and supportive strategies adapted to the stressful context. This includes home isolation, quarantines and social gathering restrictions which have evolved into significant population challenges. Home isolation, quarantines and the associated experience of loneliness should be of great concern for public mental health service providers. Loneliness has been associated with a wide range of mental health problems, interpersonal issues, substance use, and physical health conditions, including cognitive decline, and significantly elevated morbidity and mortality [48]. The findings of this study provide a rationale for an urgent call for a public health response to the mental health impacts of Covid-19. Diagnostic strategies must be developed to detect particularly vulnerable individuals. To illustrate, a study of the status of psychiatric patients in 12 countries indicated self-reported worsening of psychiatric conditions in two-thirds of the patients assessed with significantly higher scores on scales for general psychological disturbance, post-traumatic stress disorder, and depression [49]. Therapeutic strategies must be developed to reach out to individuals and population sectors affected by Covid-19. Some emergent suggestions have included counselling, befriending, referral to community resources [50] and engagement in physical activity [24, 51].

The present study offers a new slant on appropriate clinical interventions during this period with a focus on strengthening resilience factors that can moderate mental health decline. This demands a rigorous investigation of personal, family and community resilience factors that can be harnessed to address psychological distress during the pandemic. Disentangling the role of risk and resilience factors would expand knowledge of processes operating in pandemic-related psychological distress and provide important indications for treatment and prevention.

Comprehension of the psychological implications of recurrent periods of home isolation and quarantines is emerging during the predicament facing the world at present. This is further complicated by uncertainty regarding resolution of the pandemic and forced lifestyle changes. Together with these challenges, previously unknown opportunities have emerged in the new global situation for innovation, adaptation and development of therapeutic approaches anchored in a solid foundation of research.

## Data Availability

The datasets used and/or analyzed during the current study are available on Mendeley Data: Peer, Ayelet (2021), “Covid-19 Home Isolation Manuscript Data”, Mendeley Data, V1, https://doi.org/10.17632/m76jv5wkhh.1
